# Implementation of a Managed Care Programme in Germany Using the Example of the Baden-Wuerttemberg Contract on Care in the Field of Cardiology

**DOI:** 10.5334/ijic.6470

**Published:** 2022-04-29

**Authors:** Patrick Hennrich

**Affiliations:** 1 Heidelberg University Hospital, Department of General Practice and Health Services Research, DE

**Keywords:** managed care, cardiology, selective contract, integrated care, ambulatory care, Germany, implementation

## Abstract

This thesis analysed the implementation of the German medical specialists’ contract in cardiology, a managed care programme within the state of Baden-Wuerttemberg. Research focused on exploring differences between participating and non-participating physicians, their motivation to participate, actual implementation of the programme and its perceived effects. Mixed methods consisting of questionnaires and qualitative telephone interviews with cardiologists and general practitioners showed that participants were older than non-participants, participation was mainly financially driven and implementation was successful regarding medical aspects, but less so regarding patient services and communication between providers. The majority of physicians in the study perceived little to no impact of the programme on quality and efficiency of cardiology care. Still, they saw their expectations fulfilled.

## Introduction

In 2009, two German health insurers introduced the medical specialists’ contract in cardiology (cardiology programme) within the state of Baden-Wuerttemberg (approximately 11 million inhabitants). This managed care programme is the first selective contract in Germany targeted at medical specialists in ambulatory health care. As a medical specialists’ counterpart to general practitioner(GP)-centred care, it aims for efficient, high quality cardiovascular care, coordinated between medical specialists and GPs in the ambulatory sector. Key features of the programme include quality-related requirements for medical specialists, strict adherence to clinical guidelines and standardized means and regulations for communication between GPs and medical specialists. Participating physicians receive a higher reimbursement than in usual care. Patients, in turn, are promised the overall benefit of improved cardiology care as well as mainly access-related services, such as faster appointments compared to usual care [1].

The cardiology programme is the first one of its kind in Germany and subsequent selective contracts for other medical fields were designed in a similar fashion. Still, no evaluation of the cardiology programme had taken place yet. This thesis aimed to understand implementation of this selective contract and to ultimately allow for an assessment of whether and how managed care programmes can be successfully implemented in Germany and how they are implemented de-facto. The thesis examined four specific research questions: 1) Are there systematic differences between physicians who participate in the programme and those who do not? 2) What motivates physicians to participate in the cardiology programme and what are barriers? How are the initial motives and expectations of participating physicians related to the programme’s aims in this regard? 3) How are the contents of the programme actually implemented by participating medical specialists and cooperating GPs? What is the role of context factors for the implementation? 4) To what degree do participating physicians perceive effects of the programme on patient care?

## Methodology

The study was performed using a mixed-methods approach. The qualitative phase included telephone interviews with practice-based medical specialists active in cardiology care inside and outside the programme as well as GPs inside and outside GP-centred care. The quantitative phase included written questionnaires for practice-based medical specialists active in cardiology care inside and outside the programme as well as GPs in GP-centred care.

## Main results

Telephone interviews were conducted with 23 medical specialists inside and 11 medical specialists outside the cardiology programme. Furthermore, 18 GPs inside and 8 GPs outside GP-centred care were interviewed. The written questionnaire was completed by 75 medical specialists inside and 21 medical specialists outside the cardiology programme as well as 73 GPs participating in GP-centred care.

Medical specialists participating in the cardiology programme were significantly older than non-participants (on average +8.6 years in the qualitative study (95%–CI[3.49; 13.71]; t(30) = 3.44; p = 0.002), and +3.9 years in the quantitative study (95%–CI[0.49; 7.22]; t(94) = 2.27; p = 0.025). Additionally, they cooperated with significantly more GPs (on average +37 GPs (95%–CI[5.71; 68.79]; t(89) = –2.35, p = 0.021)). Participation in the cardiology programme was largely financially driven (named by 80.0% of the participating medical specialists as one of three main reasons for their participation). Care-related aspects, such as a desire to improve certain elements of patient care (named by 10.7%-33.3% of participating medical specialists) or to improve cooperation with GPs (named by 17.3% of participating medical specialists), were considered important by fewer respondents. Non-participants mainly perceived barriers to participation originating from concerns about additional administrative (66.7%) and financial burden (28.6%) in the programme [2].

Implementation of the cardiology programme was heterogeneous: Purely care-related elements of the programme, such as adherence to clinical guidelines and preferred prescription of generic medication were comprehensively implemented by participating physicians, but also by usual care providers outside of the programme. Regarding access to care for participating patients, waiting times for appointments exceeded the programme’s limits in 63.0% of the practices. Physicians identified the high numbers of participating patients as a barrier to timely access. Discrepancies were also found for cooperation- and communication-related requirements when it comes to working with GPs: Implementation on both sides varied with respect to contents and quality: Reports were transmitted significantly faster than in usual care (Fisher’s Exact Test = 8.31, p = 0.030, n = 94; Cramer-V = 0.30, p = 0.042) – meanwhile, the pre-structured report forms provided for GPs were used regularly by only about 23% of physicians. Relevant context factors that affected implementation were regional aspects and especially the structure and staffing of the individual practices [3].

Regarding effects of the cardiology programme on health care, physicians by the majority perceived vague effects or none at all. Still, 70.27% of participating physicians stated that their own expectations regarding the programme were met. For physicians who named financial aspects as a motive to participate there was a positive association between the motive and the degree to which their expectations were met (Fisher’s Exact Test = 15.11, p = 0.001, n = 73). The same goes for those who participated because they expected additional, diagnostic possibilities (Fisher’s Exact Test = 7.94, p = 0.041, n = 73).

## Implications

The thesis showed that managed care per se can be feasible in the German context. However, the results suggested that certain obstacles need to be recognized beforehand: Physicians might be motivated to participate in a managed care programme for financial reasons rather than medical reasons. This demands caution, since in a worst-case scenario, such motives can result in a focus on financial aspects at the expense of patient care [4,5]. On the other hand, higher reimbursement can also be used to extend one’s own services in patient care [6,7,8] and is known as a crucial incentive in managed care [9,10].

For actual implementation of managed care, the results showed that time-sensitive services beyond usual care require careful planning of capacities, so their availability is ensured even when there are high numbers of participating patients. Inconsistent implementation of cooperation-related aspects then suggested a low practicability of the cooperation requirements with a resulting de-facto redesign by the physicians [11,12,13,14]. Managed care that bridges the gap between general practice and specialist care can foster integration of both sides through improved communication – however, the results made it questionable whether sudden, fundamental changes of communication means, such as the introduction of pre-structured report forms, bear any additional value. Here, utilizing already existing structures (and probably introducing smaller, more gradual changes) might be preferable.

Finally, the participating physicians perceived little effect of the programme on health care. Later, this has partly been backed up by research on the outcomes of the cardiology programme: There were fewer hospitalisations and a positive effect of participation on mortality for some patients. However, the results were not clearly linked to the cardiology programme itself, since the researchers could not rule out a selection bias introduced by the physicians regarding which patients were made aware of the programme [15,16,17]. The federal agency that funded the evaluation therefore did not recommend the programme to be implemented on a larger scale [18].

These ambiguous outcomes and little differences to usual care suggest that the quality of usual cardiology care might already be high and that the cardiology programme simply does not tackle actual gaps in ambulatory cardiology care in Germany. Managed care that aims at bridging gaps in a certain field within the ambulatory sector can only reach this aim when these deficits and gaps are identified beforehand. In this regard, future programmes might want to involve patients and the public in the design phase to ensure a broader perspective on possible gaps in ambulatory care and to ensure that patients’ needs are understood and covered. In Germany, future research and similar programmes also should consider focusing on integration of care in larger regions with explicit differences in quality and availability of care that go beyond a single state, such as they still can be found between East and West Germany.
